# A Call to Action: Using and Extending Human-Centered Design Methodologies to Improve Mental and Behavioral Health Equity

**DOI:** 10.3389/fdgth.2022.848052

**Published:** 2022-04-25

**Authors:** Colleen Stiles-Shields, Caroline Cummings, Enid Montague, Jill M. Plevinsky, Alexandra M. Psihogios, Kofoworola D. A. Williams

**Affiliations:** ^1^Section of Community Behavioral Health, Department of Psychiatry and Behavioral Sciences, Rush University Medical Center, Chicago, IL, United States; ^2^Department of Psychological Sciences, Texas Tech University, Lubbock, TX, United States; ^3^College of Computing and Digital Media, DePaul University, Chicago, IL, United States; ^4^Pediatric Transplant Center, Children's Hospital of Philadelphia, Philadelphia, PA, United States; ^5^Department of Psychiatry, Perelman School of Medicine, University of Pennsylvania, Philadelphia, PA, United States; ^6^Division of Oncology, Children's Hospital of Philadelphia, Philadelphia, PA, United States; ^7^Center for Behavioral Intervention Technologies, Department of Preventive Medicine, Northwestern University Feinberg School of Medicine, Chicago, IL, United States

**Keywords:** human-centered design, equity, anti-racism, mental health, digital mental health

## Abstract

Mental health disparities directly tie to structural racism. Digital mental health (DMH), the use of technologies to deliver services, have been touted as a way to expand access to care and reduce disparities. However, many DMH fail to mitigate the persistent disparities associated with structural racism that impact delivery (e.g., costs, dependable internet access)–and may even exacerbate them. Human-centered design (HCD) may be uniquely poised to design and test interventions alongside, rather than “for,” marginalized individuals. In employing HCD methodologies, developers may proceed with a vested interest in understanding and establishing empathy with users and their needs, behaviors, environments, and constraints. As such, HCD used to mindfully address structural racism in behavioral health care may address shortcomings of prior interventions that have neglected to elevate the voices of marginalized individuals. We argue that a paradigm shift in behavioral health services research is critically needed–one that embraces HCD as a key methodological framework for developing and evaluating interventions with marginalized communities, to ultimately promote more accessible, useful, and equitable care. The current commentary illustrates practical examples of the use of HCD methodologies to develop and evaluate DMH designed with marginalized populations, while also highlighting its limitations and need for even greater inclusivity. Following this, calls to action to learn from and improve upon HCD methodologies will be detailed. Acknowledging potential limitations of current design practices, methodologies must ultimately engage representative voices beyond research participation and invest in their active role as compensated and true collaborators to intervention design.

## Introduction

Structural racism significantly impacts the reach of health services to diverse communities. For example, over 123 million Americans live in federally-designated Mental Health Professional Shortage Areas (1). Also, researchers continue to recruit generally homogenous samples (2), hindering our understanding of how to effectively address mental health concerns in diverse communities and further exacerbating disparities (3). The continued existence of structural racism in clinical science is problematic and reinforces decades-long distrust toward mental health researchers and clinicians. Accordingly, there are recent calls to acknowledge, address, and eradicate structural racism in mental health service delivery and research [e.g., (4, 5)].

Digital mental health tools (DMH), the use of technologies to deliver mental and behavioral health services, have been touted as a promising solution in expanding access to evidence-based services and reducing mental health disparities (6). There are initial data supporting the feasibility and acceptability of DMH in improving the mental health and wellbeing of individuals from diverse backgrounds [e.g., (7)]. Due to pandemic-related social distancing mandates, the use and relevance of DMH has increased rapidly (8) and aimed to mitigate the COVID-related psychiatric epidemic (9). However, many DMH fail to address the persistent disparities associated with structural racism that impact delivery. Indeed, DMH inherently requires access to digital technologies, dependable internet access, and an adequate level of literacy and experience with using technologies, all of which are common barriers to DMH engagement as reported by diverse populations (10). Further, DMH have often been designed from a top-down perspective that replicate in-person intervention procedures (11), which were primarily developed with homogenous participants, do not make use of the unique capabilities of digital health (e.g., using sensors to trigger specific interventions), and extend poorly to real world engagement for diverse users (2, 12). The field therefore stands at a critical crossroads in which to learn, improve, and generalize from design best-practices used in DMH development to address structural racism and promote equity in mental health interventions moving forward.

Namely, human-centered design (HCD) is uniquely poised to design and test interventions alongside, rather than “for” (i.e., designing without input or consideration of user needs, capabilities, or limitations), marginalized individuals who are directly impacted by structural racism in behavioral health services (13). Indeed, HCD shifts the focus simply from solving a user problem with participants [i.e., user-centered design; (14)] to, instead, better understanding the people who experience the problem. In employing HCD methodologies, developers may proceed with a vested interest in understanding and establishing empathy with users and their needs, behaviors, environments, and constraints where a product will ultimately be implemented and used. This is achieved through strategies such as interviews, focus groups, observing users, co-creation sessions with users, and rapid prototyping [see (15)]. As such, HCD used to mindfully address structural racism in mental health care may address shortcomings of prior interventions that have neglected to elevate the voices and preferences of marginalized individuals.

In this commentary, we argue that a paradigm shift in behavioral health services research is critically needed. It is imperative to embrace HCD as a key methodological framework for developing and evaluating behavioral health service interventions with marginalized communities, to ultimately promote more accessible, useful, and equitable health care. This commentary will illustrate practical examples of the use of HCD methodologies to develop and evaluate DMH designed with marginalized populations, while also highlighting its limitations and need for even greater inclusivity. Following this, calls to action to learn from and improve upon HCD methodologies will be detailed.

## Human-Centered Design

HCD involves a group of mixed-methods approaches to attempt to match a product (e.g., technology, intervention) to the contexts of its use by people in their everyday lives (16). There are a variety of HCD approaches (17–19), but they similarly focus on: 1) understanding potential users, their needs, capabilities, limitations and goals; 2) designing to address the users' concerns; and 3) evaluating the designs with the users. HCD has roots in psychological theory due to its focus on meeting cognitive capabilities and the human experience (13, 20). Namely, HCD intends to design and evaluate products alongside, rather than “for” likely end users (e.g., through strategies such as co-design workshops). It also focuses on empathy with a user's experience, something that can hold both benefits and potential problems. Indeed, as Bennett et al. (21) describe, a designer attempting to empathize and align with a blind end user by attempting to blindfold themselves while testing a product may become distracted by their own experience compared to the daily, lived experience of a blind user. As such, while HCD principles may provide a methodological solution to addressing structural racism and promoting equitable design, its history and limitations must first be acknowledged and addressed.

Despite HCD's framing as a collaborative approach with end users, power structures are insidious to its practice history (22). Indeed, the potential for the development of the following power structure is rampant in HCD: a designer, often a cisgender, heterosexual white male, attempting to “uncover” the needs of a marginalized user and/or community to create a product (23). Marginalized users and communities may often already have strong notions of what might work for them and do not need these ideas to be “uncovered” for them (24). Rather, support from those in power is needed to amplify the community's voices and implement their ideas (25). Further, end products of HCD processes have not been equitably distributed or shared in marginalized communities after they have been developed and evaluated. In sum, HCD has typically not been practiced through the lenses of equity, cultural inclusiveness, and anti-racism. We cannot promote this group of approaches as a means to promote equity in interventions without explicitly acknowledging that it has historically not been used in this way–and will not be used for equity without purposeful and continued scrutiny of its practice (22).

Moving forward, HCD methodologies may be used to promote equity, but only if actively conducted intentionally from an anti-racist perspective [i.e., the perspective that there is nothing inherently “right or wrong” with any given racial group and that racist policies and systems drive racial inequities; (26)]. While users are considered at every stage of design, explicit recommendations for considering users' full contexts must be made to fall in line with this perspective. For example, a user of a medication adherence app to support a chronic condition must also be designed within the context of potential experiences of racism within and outside the health system. Marcu and colleagues (27) demonstrate exploring such experiences from an empathy-driven design approach with youth living with human immunodeficiency virus (HIV). Similar considerations of full contexts would create opportunities for the voices of a marginalized population to be heard, but also facilitate insight into the societal and institutional factors that contribute to power imbalances that are present in both design and healthcare systems.

Multiple actions can be taken to support more inclusive HCD methodologies–both in the development of DMH and broader behavioral health interventions and services. First, intervention designs and evaluations should be conducted by diverse research teams, with an emphasis on recruiting, retaining, and promoting team members with demographic membership that is in some way representative of potential participants and end users (28, 29). There may be various barriers to doing so, including the limited number of diverse trainees within the field of psychology (30). Some ways to increase this practice and how to invest in trainees from underrepresented groups, thereby diversifying the training-to-workforce pipeline, are described in detail below. It is also important to note that, even after increasing representation of team members, researchers may need to generate a process for managing inconsistencies between team member recommendations, especially those based on diverse team members' lived experiences and evidence-based theory, which has historically been developed based on data from primarily homogenous samples (2). Emerging work on incorporating lived experience and patient-centered care with evidence-based treatments may inform such processes (31, 32). Second, research and design teams should engage in ongoing training and education in anti-racism and pertinent contextual factors [e.g., positionality practices in equity research; (33)]. Such education would promote considerations of larger contexts for users. A recent example of expanding contextual considerations is Stern et al. (34) adaptation of Bronfenbrenner's Bioecological Model to focus on Black youth development and attachment processes. Relating to this education, teams should frequently be reflecting on whether and how the design and research are serving their potential end users and practice reflexivity in recognizing when researchers are speaking for end users (4). Third, teams should collaborate with community partners to ensure that design and intervention decisions fully reflect all contexts of potential users. Doing so would likely uncover additional relevant contexts for design [e.g., Black girls' use of an intersectional lens [race and gender] to define their health and choose health-related behaviors; (35)]. To do this successfully, community partners must be given actual and active partnership throughout the design process. Indeed, rather than viewing community partners as research participants (e.g., one time participation in a usability testing session), they should be given ample agency, compensation for their time, and credit for their contributions (e.g., authorship). Collaboration with community partners may require additional resources and time, thus funding opportunities should prioritize such work. Fourth, recruitment and retention efforts for potential participants would benefit from established community-based participatory research (CBPR) methodologies [see (36)]. Action research, a family of methodologies that are responsive to a pressing need of a group while also promoting mutual learning between those facing an internal problem and outside researchers, may also complement HCD methodologies throughout the research development and recruitment stages (37). These steps are most certainly not exhaustive. We both hope and expect that means to ensure that HCD methodologies are more inclusive and are expanded well beyond this commentary.

## Narrowing the HCD Gap to Advance DMH: Case Examples

Though HCD methodologies have been successfully implemented in DMH research (38), the digital divide remains and so do the resulting health inequities in behavioral health (39). Marginalized communities are often left out and, subsequently, their needs remain unmet (40). These health disparities not only further exacerbate mental health, but impact users' engagement with DMH (41). Potential users are deserving of equitable design. Researchers must apply HCD design work inclusively to appropriately address varying needs of those underserved and underrepresented. Researchers and clinicians must thoughtfully approach their work with the unique needs of traditionally excluded populations in mind and the understanding that there are systems in place to reinforce structural and repressive racism (42). The following case examples demonstrate applications of HCD principles to designing, developing and implementing DMH work that takes into account the diverse needs of marginalized and traditionally excluded populations.

### Centering Black Men's Mental Health Needs Using Mixed Methods and Digital Tools

Noticeably absent from DMH literature is quantitative and qualitative evidence describing HCD application and principles to mental health work focused on young, Black men. Young, Black men, especially those in college, continue to be at heightened risk of experiencing mental health symptoms; however, their utilization of traditional mental health services remains low (43, 44). Disparities in mental health services utilization among minoritized populations is well-documented in the literature (45, 46), showing that Black men overwhelmingly lack adequate access to quality care. Further, men's underutilization of mental health care is not indicative of men's unwillingness to use resources but rather due to social factors and attitudinal barriers, including masculine ideology (47, 48), medical mistrust (49), and mental illness stigma (50). Creditable research has been conducted to examine and address treatment and access disparities among minoritized populations (51, 52); however, existing interventions, though successful, lack contextual and cultural relevance appropriate for attenuating the social and attitudinal barriers influencing Black men's low engagement with mental health support and the factors that stifle their uptake of digital mental health tools. Subsequently, the HCD evidence base for promoting positive mental health outcomes and equitable design work among and with this underserved population is limited.

An HCD lens is being applied to a line of research focused on reducing mental health risk among Black undergraduate and graduate men, as well as designing DMH and social media tools that promote help-seeking. The subsequent goal of this work would be to implement and test a social media-based intervention for Black men who may or may not be experiencing anxiety and depressive symptoms and wish to seek help for such concerns. Recognizing that Black men in college are traditionally and significantly underrepresented in prevention and intervention work, a mixed-methods approach was used to quantitatively elucidate factors pervasive in increasing anxiety and depression risk among Black male students and qualitatively contextualize this risk and their willingness to engage in traditional, formal health services. Over 50% of a Black male student population experienced one or more anxiety symptoms and more than 80% experienced one or more depressive symptoms; yet their utilization of traditional, in-person, counseling services remained low compared to their male counterparts (53, 54). This data provides further evidence that Black men in college are less likely to utilize formal services and underscores the importance of examining the future utility of Non-traditional avenues in mental health promotion and prevention.

Globally, more than four billion people use social media (55) and more than 95% of young people own a smartphone (56). Further, digital tools, specifically social media, are increasingly being used, especially among Black men (57–60). A 2-fold study that quantitatively assesses Black college men's social media use and qualitatively explores how social media and mobile based apps can support their mental health needs is underway. Findings from these methods will highlight key aspects associated with social media platform preferences and stress relief practices will be determined. Additionally, these studies will provide critical context for informing the development of content and themes for social media messages to promote mental health among Black men. Development of these messages will be completed using methods such as co-design workshops and usability testing sessions, which are often highlighted in HCD work as formative processes necessary in informing the design of digital tools and products that appropriately center the needs and preferences of potential users (61). The inclusion of these methods requires collaboration with Black men in college in determining mental health needs and ensuring that messages are relevant, relatable, and acceptable for men's mental health-related, help-seeking needs.

The use of multi-method approaches establishes a unique opportunity for Black men to partner with researchers to iteratively design messages that accurately address the social and attitudinal barriers most prevalent in impacting their engagement with DMH interventions. Their input provides insight and evidence into creating digital components that promote the inclusion of contextually and culturally relevant content into interventions, increasing potential users' long-term use, engagement, and accessibility (62). This application of HCD will reflect the mental health experiences of Black men and allows us to incorporate design elements and features into design that are not only relevant to Black men but will also promote tailoring and adaptation of tools for Black men and other underserved populations. This consideration is a step toward creating useful and sustainable, digital-based, mental health interventions and technologies that are empowering and equitable (63).

### Medication Taking Intervention for Diverse Adolescents and Young Adults With Cancer

Principles of HCD are being applied to develop a personalized mobile intervention for medication taking among diverse adolescents and young adults (AYA) with cancer. AYA with cancer are considered a medically underserved age cohort who experience disparities in access to developmentally-oriented cancer care (64) and experience more challenges with taking cancer-related medications than their younger counterparts (65). In addition to age, youth who identify as Black or Hispanic have demonstrated more challenges with an oral chemotherapy called 6-mercaptopurine [6-MP; (66, 67)], which is a daily medication prescribed for ~18 months to prevent an acute lymphoblastic leukemia relapse. Racial disparities with medication taking do not reflect biological differences in abilities to manage a complex disease such as cancer, they are a proxy for structural racism and other specific forms of oppression within and outside of the health system (68).

Still, adherence-promotion interventions for AYA with cancer are lacking, and those that are available have not elevated their needs and preferences. For example, one intervention involved text messaging to prompt caregivers to supervise youth as they took 6-MP (69). While this approach had promising results for adolescents within a research context, not including AYA or their caregivers in the design process may result in limited real-world effectiveness. Supervised medication taking may undermine AYA autonomy and lead to disengagement, or have feasibility challenges for families who cannot observe medication administration due to competing work and childcare demands–the latter may be especially true for families who experience economic marginalization. Indeed, many practices derived from research have fundamental design problems that limit their effectiveness. Adopting a strengths-based view of adolescence that recognizes how youth can be transformative in the intervention design process and with their own health is consistent with anti-racist scholarship on adolescence (70).

We are developing an app to help AYA take 6-MP through personalized and daily adherence support (i.e., a just-in-time adaptive intervention). Combining principles of HCD and anti-racist practices, we have approached intervention development with the following guiding principles: 1) systemic racism and other forms of oppression influence treatment behaviors and oncology care, and can influence (implicitly or explicitly) researcher and participant perceptions; 2) AYA are the experts on their own experiences; 3) AYA are capable of engaging in the research process as collaborators (not only participants) when our research team is successful in making the research process inclusive; 4) AYA experiences with medications are not monolithic or “one-size-fits-all”; and 5) design is not a linear process and requires iterative cycles. Within this work, researchers deeply reflect on positionality and practice reflexivity in recognizing how their social positions can influence the research (including the inherent power dynamic that exists between the researcher and the participant). For example, researchers maintain ongoing vigilance for the urge to speak for participants.

The British Design Council's Double Diamond Framework has guided our HCD methodological approach (71). This process involves a combination of divergent (deep exploration about an issue and ideation of possible solutions) and convergent thinking (consolidating insights to solidify the type of solution and its design) across four iterative steps–discover, define, develop, and deliver. Fundamental to the discovery stage is a good understanding of AYA with cancer and their needs for an intervention. Toward this goal, we employed mixed-methods (e.g., qualitative interviews with AYA and their caregivers and oncology providers; quantitative surveys; ecological momentary assessment; collaborative brainstorming sessions) to understand current medication taking patterns, practices, needs and preferences for intervention, and contextual determinants (72, 73). Synthesis of these formative studies led us to define our solution as a personalized DMH.

Now, we are in the process of co-developing the intervention (e.g., app design features, message content, engagement strategies, how to link the app to other individuals and systems that impact adherence) with AYA. We formed a research advisory panel of AYA with a history of cancer where advisors serve as paid research consultants to ideate intervention content with the research team. Four advisors, either self-nominated or nominated by a member of their oncology team, were trained using Family, Youth, and Research Education (FYREworks), an interactive, web-based training that prepares youth to collaborate with researchers. We will engage with the advisors throughout the iterative intervention development process, most recently with a focus group that applied a “Blue Sky Thinking” design strategy to generate creative solutions for medication taking without imposed limits. In this activity, advisors were presented with two AYA patient stories or personas, and shared ideas for improving each patient's medication adherence. For example, advisors recommended linking medication-taking to an AYA's personal passions and goals to motivate adherence, as preventing a cancer relapse seemed too distal and focused on the AYA as a patient rather than a person. AYA end users have also developed intervention content, such as self-creating TikTok videos designed to promote adherence.

We believe that partnering with AYA in the design process in this manner is an important first step toward creating an inclusive and engaging tool for medication taking. However, an app will not completely address systemic barriers to adherence [e.g., prohibitive costs of care, patient caution about the prescription due to historical medical abuse of Black populations; (74)], nor is this intervention focused exclusively on youth who have experienced racism and other specific forms of oppression.

## Future Outlook and Recommendations

### Invest in the Career Achievement and Advancement of Marginalized HCD Researchers

To ensure that HCD practices are conducted by and with diverse research teams and principal investigators, the recruitment, retainment, and promotion of researchers with marginalized identities must be an institutional priority. To achieve this goal, investing in marginalized youth cannot start early enough. Examples of such investment is the Rush Education and Career Hub (REACH), whose mission is to provide STEM training and exposure to underrepresented youth to support their academic and professional potentials from “cradle to career” (75), and the Minority White Coat Foundation, which provides mentorship, training, and support to increase the number of minorities in healthcare positions, including behavioral healthcare positions (76). Models developed by groups such as REACH and the Minority White Coat Foundation may be expanded to other institutions and supported by established researchers (e.g., provide mentoring, share data and experiences) and funding sources (e.g., funding opportunities to support HCD methodology training, mentorship, opportunities for student-led research initiatives). To extend pathway programs beyond the start of the career, the promotion of diversity, equity, and inclusion (DEI) efforts must not be placed at the feet of established researchers with marginalized identities. Indeed, such work is often uncompensated and interferes with the advancement of their own work (77). Rather, institutions should require annual mandatory curricula on anti-racism and inclusive research practices. Annual budgets for this education must also include compensation for time and effort of presenters. Finally, institutions should focus on promoting the work of HCD researchers with marginalized identities and encourage their work as compensated consultants. Due to a lack of exposure to HCD training, behavioral health specialists will often require external consultation to implement HCD methodologies into their own work. Creating pathways within institutions for HCD researchers to offer compensated consultations to behavioral health collaborators will: 1) promote the broader use of inclusive HCD methodologies for behavioral health intervention development; 2) appropriately compensate HCD researchers for their time and expertise; and 3) increase the likelihood of representation of experts with marginalized identities to behavioral health trainees and early career professionals.

### Create and Recognize Active Role of Community Voices in HCD

The use of HCD methodologies must invest in creating active and compensated roles for community members in intervention design. Indeed, community members must not be viewed as “participants” but as part of the HCD research team itself. A key factor in this approach would be the practice of reflexivity and acknowledging the power imbalances that exist between and among all parties involved throughout the research process (78). Additionally, teams should collaborate with community partners and organizations to identify appropriate and preferred compensation (amount, type), venues through which to be engaged, and ways the research and its findings can invest back into the lives of community members (36, 79). Community partners should also be formally acknowledged (e.g., authorship) and welcomed to opportunities for dissemination (e.g., co-presenting at conference symposia).

### Increase Funding Opportunities for HCD Research, Training, and Resources

Funding partners are needed to facilitate open access to HCD methodologies and promote their use. Federal and Non-federal funding mechanisms may provide opportunities to support this work, invest in the advancement of marginalized researchers, and promote service design aimed at systems-level changes in behavioral healthcare. Review boards are often siloed within their subspecialties and do not include experts in HCD methodologies, nor do they include individuals representative of the communities with whom the projects are intended to reach. We propose that bodies charged with disseminating funding for behavioral health research seek partnerships outside the behavioral health care field, including experts in HCD and community partners as discussed above. Additionally, funding bodies may consider HCD as a core component of any effort to increase health equity, thereby requiring researchers to include aspects of the HCD framework in their proposed work. For those specifically engaged in health disparities work, such requirements will serve to prevent the already documented phenomenon of white researchers without adequate training or understanding of these disparities capitalizing on funding opportunities without including their colleagues and community members of color (80).

To increase the use of HCD, training in this methodology needs to be accessible as well. Although experts in digital health may be familiar with user-centered design, it is imperative that they consider the more inclusive HCD framework discussed in this commentary. Though some clinical researchers are embracing HCD [e.g., (27)], most have not received formal training in HCD nor have natural pathways to form partnerships with human computer interaction researchers and human factors engineers. Providing access to training and creating a visible network of diverse scholars engaged in HCD, especially within the DMH space, will be key for the continued training of our mental health research workforce and ongoing promotion of inclusivity in our research efforts.

### Apply HCD Practices to Address Structural Racism in Behavioral Healthcare

HCD practices of specifying the context of use may be harnessed to explicitly explore, identify, and address the role and impact of structural racism in behavioral healthcare through targeted and measurable means. First, interdisciplinary collaborations are needed and appropriate for determining, examining, and addressing the fundamental factors that impact underlying disparities and inequities which reinforce population health outcomes as well as policy. Second, while a primary focus of this commentary has been on employing HCD practices to co-develop DMH with community members, HCD can also be applied by clinicians and within clinics to develop: (1) DMH service protocols (i.e., how it is delivered in clinical care); and (2) implementation plans [e.g., training providers in cultural humility to recognize the limitations of DMHs and make adaptations; (81, 82)]. Indeed, clinicians and clinics may benefit from the structure of HCD methodologies as they develop service protocols, as integrating new interventions into practice poses challenges–especially for digital interventions, which differ from traditional in-person delivery. The inclusion of implementation plans in the use of HCD methodologies is also likely key, as research-based trials demonstrate poor uptake and engagement in real-world settings and there are few examples of successful digital intervention integration in existing care settings (83). Further, without designing for implementation, clinics run the risk of reinforcing inequities (e.g., bias in how tools are distributed) that DMH are purported to address [e.g., increasing access; (82)].

It is of note that while we propose that HCD methodologies may be used with intention to design DMH and broader behavioral and mental health interventions, these efforts cannot address inequities and racism alone. Systems-level changes are desperately needed, from a larger and more diverse workforce of behavioral health professionals to health care coverage for mental health services.

## Conclusions

It is necessary to incorporate HCD methodologies into DMH work to achieve mental and behavioral health equity. However, changes in the practice of engaging in HCD methodologies are merely the start of changes needed to promote equity, cultural inclusiveness, and anti-racism. Steps detailed in this commentary are most certainly not exhaustive of what should and could happen moving forward. We recognize that there are critiques of HCD methods for the promotion of equity and inclusiveness and we believe that ongoing research and initiatives that we describe will lead to the growth of HCD methods for addressing the complex problem of improving mental and behavioral health equity. Advances in HCD methodology will be needed to address these problems, but HCD is an approach that can be used to improve equity in the development of DMH and broader forms of behavioral interventions.

## Data Availability Statement

The original contributions presented in the study are included in the article/supplementary material, further inquiries can be directed to the corresponding author.

## Author Contributions

CS-S: conceptualized the commentary. All authors contributed to the drafting of the manuscript and approved the final version. All authors contributed to the article and approved the submitted version.

## Funding

This work was supported by grants from the National Institutes of Health (K08 CA241335; K08 MH125069; T32 MH115882-03; K08 MH112878).

## Conflict of Interest

The authors declare that the research was conducted in the absence of any commercial or financial relationships that could be construed as a potential conflict of interest.

## Publisher's Note

All claims expressed in this article are solely those of the authors and do not necessarily represent those of their affiliated organizations, or those of the publisher, the editors and the reviewers. Any product that may be evaluated in this article, or claim that may be made by its manufacturer, is not guaranteed or endorsed by the publisher.
